# In vitro study on antioxidant and lipid-lowering activities of tobacco polysaccharides

**DOI:** 10.1186/s40643-024-00729-9

**Published:** 2024-01-23

**Authors:** Shuaishuai Chang, Xiao Lei, Qiang Xie, Mingjin Zhang, Yuangai Zhang, Jiaxin Xi, Jiyou Duan, Jian Ge, Fuzhao Nian

**Affiliations:** 1 China Jiliang University School of Life Sciences, Hangzhou, 310018 China; 2 Luzhou Branch of Sichuan Tobacco Company, Luzhou, 646000 China; 3 Yongren Branch of Chuxiong Company of Yunnan Tobacco Company, Chuxiong, 651400 China; 4 Yunnan Agricultural University School of Tobacco, Kunming, 650201 China

**Keywords:** Tobacco polysaccharide, Physical and chemical properties, In vitro antioxidant, Lipid lowering

## Abstract

Tobacco polysaccharides were extracted by hot water extraction, and purified and separated using DEAE-52 cellulose chromatography columns, and three purified polysaccharide fractions, YCT-1, YCT-2, and YCT-3, were finally obtained. The physicochemical properties of the three fractions were analyzed by ultraviolet spectroscopy, high-performance liquid chromatography and high-performance gel chromatography. The in vitro antioxidant activity of tobacco polysaccharides was compared among different fractions by using DPPH radical, hydroxyl radical scavenging assay and potassium ferricyanide method. The in vitro hypoglycemic activity was compared using α-amylase and α-glucosidase activity inhibition assay. And the in vitro hypolipidemic activity were investigated by using pancreatic lipase activity inhibition assay and HepG-2 intracellular lipid accumulation assay. All the results showed that the constituent monosaccharides of the three tobacco polysaccharide fractions were similar, but the molar percentages of each monosaccharide were different. The average molecular weights of the three components were 27,727 Da, 27,587 Da, and 66,517 Da, respectively, and the scavenging activities on DPPH radicals and hydroxyl radicals were at a high level with good quantitative-effect relationships. The reducing power were much lower than that of the positive control VC, and the three polysaccharide fractions had a weak inhibitory ability on α-amylase activity, but showed excellent inhibitory ability on α-glucosidase and pancreatic lipase activity. In addition, the results of cellular experiments showed that all three fractions were able to inhibit lipid over-accumulation in HepG-2 cells by increasing the mRNA expression levels of PPAR-α, CPT-1A, and CYP7A1 genes, and the tobacco polysaccharide YCT-3 showed the best effect. The mechanism by which YCT-3 ameliorated the over-accumulation of intracellular lipids in HepG-2 cells was found to be related to its influence on the expression of miR-155-3p and miR-17-3p in the exosomes of HepG-2 cells.

**Graphical Abstract:**

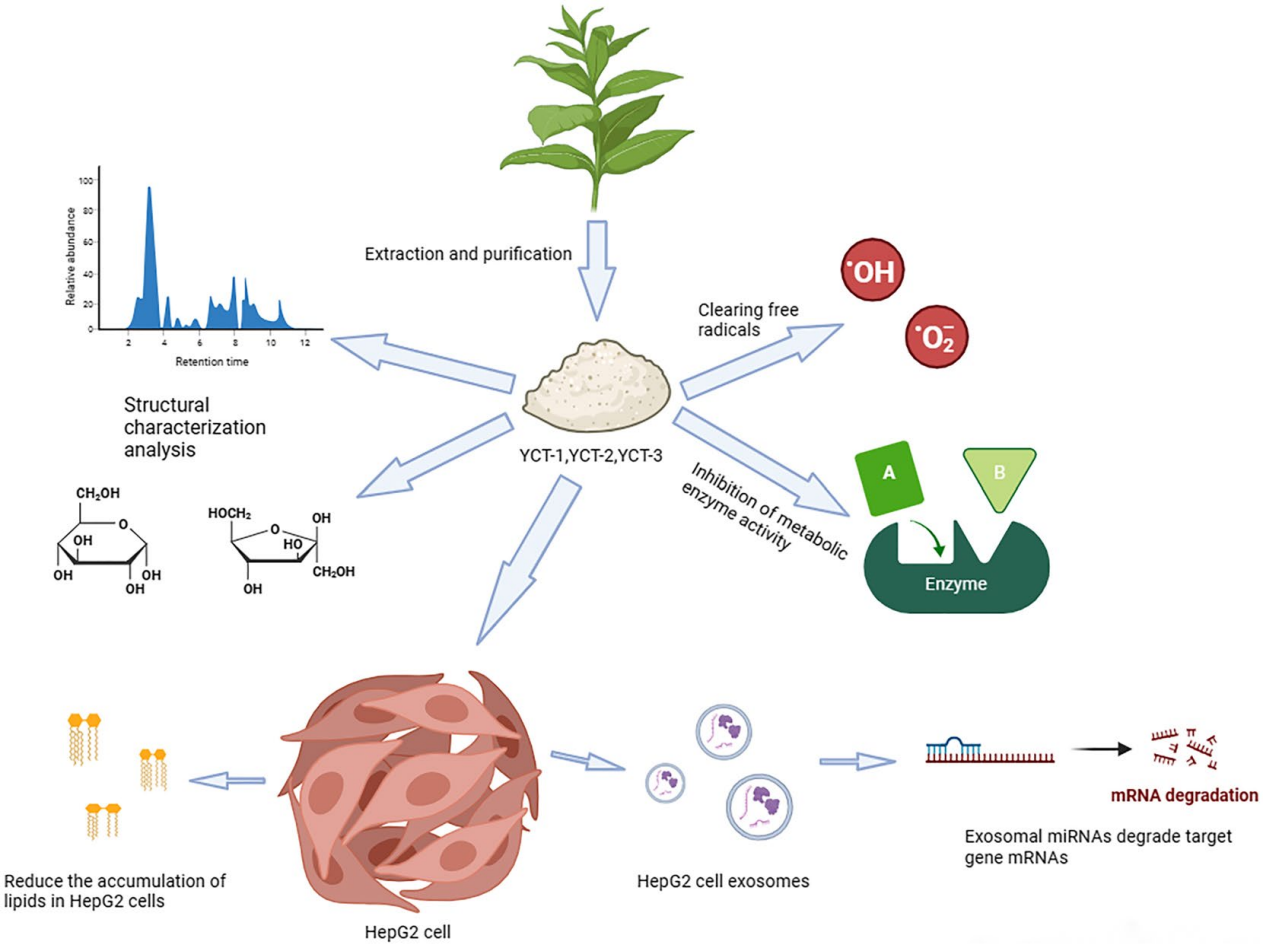

## Introduction

Tobacco (Nicotiana tabacum) is native to South America and an important cash crop in China (Wang et al. 2023). Tobacco is primarily used to make cigarette products, and a large part of the national financial tax revenue comes from the tobacco industry, which fully reflects the huge economic value of tobacco. China is a vast country with more than 3 million square hectares of land available for tobacco cultivation, making it the world’s largest producer and consumer of tobacco. During tobacco production and processing, a large amount of tobacco waste is produced every year, such as tobacco stalks, moldy tobacco, and residual tobacco, which is not up to the requirements of the raw materials for cigarette production of inferior tobacco (Xieyang et al. 2022; Xiaoping et al. 2020). At present, most of the tobacco waste is disposed by discarding, direct burial, or simple incineration, which not only results in the inefficient utilization of tobacco resources, but also causes serious environmental pollution. Therefore, the effective utilization of these tobacco wastes can result in huge economic benefits, good social benefits, and ecological benefits (Bit 2005). In addition, fully utilizing and developing tobacco resources is a topic that the tobacco industry as well as the natural resources development and utilization industry are paying keen attention to.

Research has shown that tobacco leaves contain more than 3000 kinds of natural chemical substances, including alkaloids, proteins, amino acids, and sugars, and more than 40 kinds of organic acids (Changbin et al. 2022; Dongxian et al. 2021). Tobacco waste has a variety of chemical components, mainly containing nicotine, cannabinol, chlorogenic acid, pectin, xylose, pigment, cellulose, lignin, etc. Among them, nicotine, cannabinol, chlorogenic acid, and other active substances have been proven to have good antioxidant and antibacterial efficacy, which can be used in the development of biopesticides, antibacterial soaps, and other products. Meanwhile, they can also be used as a raw material for medicine (Tao 2021; Mi et al. 2005). In addition to the extraction of important compounds from tobacco waste, it can also be used to produce compost, activated carbon, and biomass fuels (Junling et al. 2022; Jinbang et al. 2022; Wang et al. 2020a). Polysaccharides are natural macromolecular compounds consisting of multiple monosaccharides linked by glycosidic bonds, which play a key role in cellular recognition and information transfer. Polysaccharides are also a class of non-specific broad-spectrum immunomodulators and important living substances that are widely involved in a variety of life activities (Yi-Xin et al. 2023; Saifeng et al. 2023; Zhigao et al. 2023). Recent studies have shown that the application of plant-derived polysaccharides in immunomodulatory, antitumor, antiviral, antioxidant, hypoglycemic, and hypolipidemic aspects is promising (Xuan et al. 2023; Ruiwu et al. 2023; Liu et al. 2023; Yunpeng et al. 2023). It also has a high research value in the field of modern medicine and functional foods (Chenchen et al. 2022; Chunyan et al. 2022; Yang 2022). Tobacco polysaccharides, as a class of natural products in tobacco, have a broad developmental potential and numerous functional activities to be explored. However, studies on the antioxidant activity, inhibition of glycolipid-metabolizing enzymes, and lipid-lowering activity of tobacco polysaccharides have not been reported yet.

In this study, tobacco polysaccharides were extracted by hot water and purified by DEAE-52 cellulose columns, and the in vitro antioxidant activities of the three tobacco polysaccharide fractions obtained by purification were also investigated. The in vitro hypoglycemic and hypolipidemic activities of the three fractions were explored by α-amylase, α-glucosidase, and pancreatic lipase activity inhibition assays. In addition, we utilized oleic acid–induced HepG-2 cells to establish a cellular hyperlipidemia model, evaluated the hypolipidemic activity of the three fractions, and revealed their regulatory mechanisms on lipid metabolism abnormalities. Therefore, the abovementioned studies can provide a new perspective for the comprehensive utilization of tobacco resources and alleviation of environmental pollution, as well as theoretical foundation and experimental technology for the development of new plant-derived functional foods from tobacco polysaccharides.

## Materials and methods

### Materials

Dried tobacco was provided by the College of Tobacco, Yunnan Agricultural University. The Bradford method protein concentration assay kit and 100 × penicillin–streptomycin were purchased from Sole bro Biotechnology Co. Ltd. HepG-2 cells were purchased from the Cell Resource Center of Shanghai Institutes for Biological Sciences, Chinese Academy of Sciences. TC and TG assay kits were purchased from Thermo Fisher, USA. BCA protein assay kits were purchased from Biyuntian Biotechnology Co. Synthesis SuperMix kits were purchased from Thermo Fisher, USA. RNase-free DNase set kits were purchased from Qiagen, Germany.

### Methods

#### Preparation of tobacco polysaccharides

The dried tobacco leaves were crushed and passed through a 60-mesh screen to collect the smoke powder. The tobacco powder was added with anhydrous ethanol (solid–liquid ratio of 1:4) and stirred in a water bath at 55 ℃ for 4 h. Then, all the precipitates were collected and dried. After pretreatment, 50 g of tobacco powder was added with 750 mL of distilled water and stirred in a water bath at 65 ℃ for 4 h, and the supernatant was collected after centrifugation. The tobacco powder was extracted two times in the same way, and the two extracts were combined and subjected to an ice bath for 2 h. After the ice bath, the extracts were filtered to remove starch impurities. 0.1 times of the volume of the filtrate with a mass fraction of 10% trichloroacetic acid solution into the filtrate was added, stirred with an electric mixer for 2 h, stand at room temperature for 1 h, centrifuged for 10 min at a speed of 4000 r/min to remove the protein precipitates, and then added D101 macroporous resin with a volume fraction of 2.5% into the filtrate. An electric mixer was used to stir for 2 h to make the polysaccharide solution fully in contact with the macroporous resin, and the pigment impurities in the polysaccharide were removed by the adsorption of the macroporous resin on the pigment. After stirring, the resin was filtered and removed, and the filtrate was concentrated by using a rotary evaporator, added with anhydrous ethanol four times the volume of the filtrate, and precipitated at 4 ℃ for 12 h. The precipitated polysaccharides were collected by centrifugation, redissolved in ultrapure water, dialyzed for 72 h, and vacuum freeze dried to obtain tobacco crude polysaccharides. The yield of crude polysaccharide from tobacco was 4.11%.

The crude polysaccharide solution of tobacco with a concentration of 20 mg/mL was prepared, and the sample was loaded onto the DEAE-52 cellulose column. Then, the polysaccharide solution was eluted with distilled water, 0.1 mol/L NaCl solution, and 0.3 mol/L NaCl solution. After eluting with each concentration of eluent, 40 tubes of eluent were collected, with each tube measuring 8 mL, and the concentration of tobacco polysaccharides in these tubes was detected by using the phenol-concentrated sulfuric acid method. The eluted fraction with the highest concentration was collected and concentrated by using a rotary evaporation apparatus. After concentration, dialysis was performed for 72 h. After dialysis, vacuum freeze drying was performed to obtain three purified tobacco polysaccharides, namely, YCT-1, YCT-2, and YCT-3. The yields of YCT-1, YCT-2, and YCT-3 were 0.91%, 0.83%, and 0.88%, respectively.

#### Analysis of monosaccharides composition

The monosaccharide composition of YCT-1, YCT-2, and YCT-3 was analyzed by using 1-methoxy-2-propionyl propionate (PMP) derivations and the HPLC–UV method. The solution was sealed with nitrogen and heated at 121 °C for 2 h. Then, 1 mL of the supernatant was dissolved in methanol and dried with nitrogen, and this process was repeated several times. In addition, 1 mL of 0.3 mol/L NaOH solution was added to dissolve the precipitate. Then, 0.5 mL of the PMP methanol solution was added to the abovementioned solution, and then the mixture was heated at 80 ℃ for 2 h. Hydrochloric acid was added to neutralize the precipitate, and chloroform was added to extract the precipitate three times. PMP was removed by chloroform, filtered via a 0.45 µm microporous membrane, and then used for HPLC analysis. The chromatographic conditions were set as follows: column C18 (250 mm × 4.6 mm, 5 µm); detection wavelength of 250 nm; column temperature of 30 ℃; flow rate of 1 mL/min; injection volume of 10 µL; mobile phase A, 100 mmol/L sodium phosphate buffer; mobile phase B, acetonitrile.

#### Relative molecular mass detection

The molecular weight of YCT-1, YCT-2, and YCT-3 was analyzed by high-performance gel chromatography. 10 mg/mL sample solution was prepared and filtered by using a 0.45 µm microporous filter membrane. The chromatographic spectrometer was set as follows: Waters 1525; column, PL aquqgel-OH MIXED 8 µm; mobile phase, 0.2 mol/LNaNO3, 0.001 mol/LNaH2PO4; flow rate, 1 mL/min; column temperature, 30 °C.

#### DPPH free radical scavenging ability

Five milligrams of DPPH was dissolved in anhydrous ethanol and quantified using a 50 mL volumetric flask. Then, VC (positive control), YCT-1, YCT-2, and YCT-3 were weighed and prepared at concentrations of 0.5, 1.0, 2.0, 3.0, and 4.0 mg/mL. The absorbance (A value) was measured at 517 nm, and the A value of each sample solution was measured three times in parallel (An et al. 2020; Ran et al. 2017).

DPPH radical scavenging rate (%) = [(1 − (*A*_*i*_ − *A*_*j*_)/*A*_0_)] × 100%, where *A*_*i*_ is the *A* value of the sample solution mixed with DPPH solution, *A*_*j*_ is the *A* value of the sample solution, and *A*_0_ is the *A* value of the DPPH solution.

#### Hydroxyl radical scavenging ability

Certain amounts of VC, YCT-1, YCT-2, and YCT-3 were weighed and prepared at concentrations of 0.5, 1.0, 2.0, 3.0, and 4.0 mg/mL. Equal amounts of each sample solution and VC solution were pipetted into a 1.5 mL stoppered tube, which were in turn added with 0.009 mol/L ferrous sulfate solution, 0.009 mol/L salicylic acid–ethanol solution, and 0.006 mol/L hydrogen peroxide solution, mixed well, and reacted at 37 ℃ for 45 min. Then, the A values were measured at 510 nm using an enzyme marker. Each sample was measured three times in parallel (Chu et al. 2015).

Hydroxyl radical scavenging rate (%) = [1 − (*A*_*i*_ − *A*_*j*_)/*A*_0_)] × 100%, where *A*_*i*_ is the value of A with the addition of sample solution and ferrous sulfate, salicylic acid–ethanol, and hydrogen peroxide solution; *A*_*j*_ is the value of *A* without the addition of hydrogen peroxide solution; and *A*_0_ is the value of *A* without the addition of a sample solution.

#### Reduction power

YCT-1, YCT-2, YCT-3, and VC were prepared at concentrations of 0.5, 1.0, 2.0, 3.0, and 4.0 mg/mL. Equal amounts of different concentrations of sample solution and VC solution were mixed well with phosphate buffer (0.2 mol/L, pH = 6.6) and K_3_Fe (CN)_6_ (0.1%, m/v). The mixture was reacted at 50 ℃ for 20 min, and then trichloroacetic acid solution (10%, m/v) and FeCl_3_ solution (0.1%, m/v) were added and mixed well. Afterward, the absorbance was measured at 700 nm. Each sample was measured three times in parallel, and the reducing power was calculated by using the following equation (Liu et al. 2021; Yan 2021).

Reducing power = *A*_*i*_ − *A*_*j*_, where *A*_*i*_ is the absorbance value of the sample group, and *A*_*j*_ is the *A* value of the absorbance of the interference group (distilled water instead of FeCl_3_ solution).

#### α-amylase inhibition experiment

After incubating for 10 min under shade, the reaction was started by adding 1% soluble starch solution. The reaction was terminated by adding DNS solution immediately after 10 min, heated in a water bath at 100 °C for 10 min, and then cooled at room temperature. Then, distilled water was added into each tube, and the absorbance A value was measured at 540 nm. The blank group was replaced with deionized water, and acarbose was used as a positive control; each sample was measured three times in parallel (Li et al. 2022; Xiao et al. 2021; Wang et al. 2022).

The inhibition rate was calculated by using the following formula: α-amylase inhibition rate (%) = (*A*_0_ − *A*_1_)/*A*_0_ × 100%, where *A*_0_ refers to the blank group (pure water instead of sample), and *A*_1_ refers to the sample group.

#### α-glucosidase inhibition assay

Equal amounts of different concentrations (0.5, 1, 2, 3, and 4 mg/mL) of YCT-1, YCT-2, YCT-3, and acarbose solutions were pipetted into a 1.5 mL stoppered tube, and then 0.2 U/mL of α-glucosidase solution was added and mixed well. The reaction was incubated for 10 min at 37 °C, and then 5 mmol/L of *p*-nitrophenol β-glucoside was added to the mixture. The reaction was continued for 30 min at 37 °C. Finally, the reaction was ended by adding 0.1 mol/L of Na_2_CO_3_ solution, and the absorbance was measured at 405 nm (Li et al. 2023; Shu-ship et al. 2014).

The α-glucosidase inhibition rate was calculated as α-glucosidase inhibition rate (%) = (*A*_0_ − *A*_1_)/*A*_0_ × 100%, where *A*_0_ is the blank group (pure water instead of sample), and *A*_1_ is the sample group.

#### Pancreatic lipase inhibition

YCT-1, YCT-2, YCT-3, and orlistat were all prepared at concentrations of 0.5, 1, 2, 3, and 4 mg/mL. Equal amounts of different concentrations of sample solutions were added to the stoppered tube, and then 50 mmol/L phosphate buffer (pH 8.0) and 100 μL of 10 mg/mL pancreatic lipase solution were added in turn, mixed, and preheated at 37 °C for 10 min. Subsequently, 100 μL of 0.5 mmol/L 4-nitrophenyl laurate substrate solution was added, mixed, and incubated at 37 °C. After centrifugation, the supernatant was removed, and the absorbance of the supernatant was measured at 405 nm. Orlistat was used as a positive control, and each group of samples was measured three times in parallel (Shu-ship et al. 2014; Shi et al. 2008; Chen et al. 2010).

The inhibition rate of pancreatic lipase by samples was calculated as follows: pancreatic lipase inhibition rate (%) = [1 − (*A*_3_ − *A*_4_)/(*A*_1_ − *A*_2_)] × 100%, where *A*_1_, *A*_2_, *A*_3_, and *A*_4_ are the blank group (distilled water instead of sample), blank background group (distilled water instead of sample and distilled water instead of pancreatic lipase), sample group, and sample background group (distilled water instead of the absorbance of pancreatic lipase), respectively (Zhang et al. 2015; Di et al. 2021).

#### HepG-2 cells culture and CCK-8 detection

0.141 g of oleic acid was accurately weighed and dissolved in NaOH solution at 0.1 mol/L. After reaction at 70 °C for 30 min and cooling to room temperature, the solution was filtered to remove bacteria through a microporous filter membrane, and a 0.1 mol/L sodium oleate solution was obtained. Then, 10 mmol/L oleic acid was prepared by using PBS and diluted with a DMEM blank culture medium before use (Zhang et al. 2015). In addition, the solution containing YCT-1, YCT-2, and YCT-3 was prepared by using PBS at a concentration of 2 mg/mL, de-bacterized by using a microporous membrane, and diluted with a DMEM blank culture medium before use.

HepG-2 cells were cultured with 10% fetal bovine serum and 1% penicillin–streptomycin solution in 5% CO_2_ at 37 °C. After the density of HepG-2 cells exceeded 80%, the experiment could be performed at the logarithmic growth stage of cells (Lin et al. 2019). Then, the cells were seeded into 96-well plates with a cell density of 1 × 10^5^ cells/mL, and oleic acid solution (0.1, 0.15, 0.2, 0.25, and 0.3 mmol/L) or YCT-1, YCT-2, and YCT-3 solutions (50, 100, 200, 500, and 1000 μg/mL) were added to each well. In addition, the CCK-8 method was used to screen the appropriate concentration of oleic acid. After discarding the excess culture medium, 10 μL of CCK-8 was added into each well and incubated for 2 h. Then, the absorbance of each well was measured at 450 nm, and cell viability (%) was calculated in accordance with the following formula: Cell viability (%) = [(*A*_spiked_–*A*_blank_)/(*A*_0 spiked_ − *A*_blank_)] × 100%,

where *A*_spiked_ is the absorbance of wells with a CCK-8 solution and drug solution, *A*_blank_ is the absorbance of wells with a culture medium and CCK-8 solution without a drug solution, and *A*_0 spiked_ is the absorbance of wells with a CCK-8 solution without a drug solution.

#### Oil red O/hematoxylin staining and lipid accumulation rate

HepG-2 cells with a cell density of 1 × 10^5^ cells/mL were seeded in 24-well plates, and the culture medium was discarded after 24 h. A complete medium was added into the control group; 0.2 mmol/L oleic acid solution was added into the model group; 0.2 mmol/L oleic acid and 500 μg/mL of YCT-1, YCT-2, and YCT-3 solution were added into the experimental sample group. After 24 h, the culture medium in each well was discarded and washed two times with PBS, and each well was fixed with a cell fixing solution for 1 h and washed two times with sterile water. Then, 60% isopropyl alcohol was added, and then oil red staining solution was added. The cells were stained for 20 min. After rinsing with sterile water three times, Mayer hematoxylin staining solution was added for 2 min, and then the cells were rinsed with tap water. After rinsing, appropriate amount of distilled water was added into each well, and the cells were observed under a microscope. After observation, each well was washed two times with distilled water, and 200 μL of isopropanol was added. Then, the absorbance was measured at 492 nm, and the lipid accumulation rate was calculated in accordance with the following formula: (Di et al. 2020).

$${\text{Lipid accumulation rate }} = {\text{ OD}}_{\text{experimental group}} /{\text{ OD}}_{\text{model group}} \times { 1}00\%$$

#### Measurement of TC, TG, HDL-c and LDL-c in HepG-2 cells

After treatment using the abovementioned methods, the cells were lysed and collected. The TC, TG, HDL-c, and LDL-c contents of each group were calculated in accordance with the kits’ operational guidance (Ju et al. 2019; Shaolin et al. 2017; Qianhua et al. 2023).

#### RT-qPCR

After the cell culture was completed, the cells were collected using the TRIzol Plus RNA Purification Kit (Thermo Fisher), and the SuperScript III First-Strand Synthesis SuperMix for qRT-PCR (Thermo Fisher) was used for total RNA extraction and first-strand cDNA synthesis in accordance with the kit instructions. The primers were designed by Primer Premier 6.0 and Beacon designer 7.8 and synthesized by Biotech Bioengineering (Shanghai) Co. qPCR was performed using the Power SYBR Green Master Mix (Table 1). The reaction system was set as follows: Power SYBR Green Master Mix, 10.0 μL; upstream and downstream primers, 0.5 μL; cDNA template, 1.0 μL. The reaction conditions were set as follows: pre-denaturation at 95 ℃ for 1 min; denaturation at 95 ℃ for 15 s, annealing extension at 63 ℃ for 25 s. In addition, 40 cycles were performed. Each sample was repeated three times, and the relative expression levels of each gene were statistically analyzed as 2^(Ct internal reference gene−Ct target gene)^.

#### Extraction and characterization of HepG-2 cell exosomes

HepG-2 cells with a cell density of 1 × 10^5^ cells/mL were seeded into 75 mL cell flasks for 24 h. The excess culture medium was discarded, and a complete medium with free exosomes was added into the control group. In addition, 0.2 mmol/L oleic acid solution was added into the model group, and 0.2 mmol/L oleic acid solution and 500 μg/mL of YCT-3 solution were added into the experimental sample group for 24 h. Finally, the culture medium from HepG-2 cells was collected and centrifuged at 10, 000 rpm for 30 min to remove cellular debris and impurities, and then the supernatant was discarded and centrifuged using a super-centrifuge (L-100XP, Beckman, USA) at 100, 000*g* for 90 min. At the end of centrifugation, the supernatant was discarded, and then the precipitate was re-suspended by adding 100 μL of PBS. Then, the re-suspended precipitate was centrifuged again for 90 min at 100, 000*g*. After centrifugation, the supernatant was discarded, and the precipitate was re-suspended again with 200 µL of PBS. The extracted cell exosomes were then identified by using a transmission electron microscope (TEM, HT7700, HITACHI, Japan), and the particle-size distribution was analyzed using a flow nano-analyzer (N30E, Xiamen Flow Biotechnology Co, Ltd.) (Di et al. 2021, 2020; Lin et al. 2019; Ju et al. 2019). Furthermore, exosome proteins were detected by Western blotting.

**Table 1:** Primers sequences used for Real-Time PCR

Gene	Genbank Accession	Primer sequences (5' to 3')	Amplicon size (bp)
GAPDH	NM002046.5	CCATGACAACTTTGGTATCGTGGAA GGCCATCACGCCACAGTTTC	107
PPAR-α	CR456547.1	CCCTCCTCGGTGACTTATC	111
		GTAATGATAGCCTGAGGCCTTGT	
CYP7A1	NM_000780.4	CCTCCAGTCTCCTCTAACTCA	126
		GTCCCGCCTTGTAAGATCTCT	
CPT1A	NM_001876.4	CACATTCAGGCAGCAAGAGC	121
		CGGAGCAGAGTGGAATCGT	

#### RT-qPCR determination for miRNAs levels in HepG-2 cells exosomes

The molecular basis of the interactions between miR-155-3p and its target gene PPAR-α mRNA, or miR-17-3p and its target gene CYP7A1 mRNA, was predicted using Target Scan (https://www.targetscan.org/vert_80/). In brief, human was selected as the species, and PPAR-α and CYP7A1 genes were entered in the corresponding boxes. The prediction results will be displayed in a new window after clicking the submit button. The miRNAs were synthesized by reverse transcription using Super Script III reverse transcriptase (Thermo Fisher, USA). Quantitative PCR primers were designed using Primer Premier 6.0 and Beacon designer 7.8 and then synthesized by Shenggong Biotech (Shanghai) Co., Ltd. The primer sequences and RT-qPCR conditions are shown in Tables 2, 3, 4. RT-qPCR was performed using the Power SYBR Green PCR Master Mix (Applied Biosystems, Inc., USA) and CFX384 multifunctional Real-Time PCR instrument (Bio-Rad, USA). Each sample was repeated three times, and the relative expression level was utilized to perform 2^−ΔΔCt^ statistical analysis (Shaolin et al. 2017; Qianhua et al. 2023).

**Table 2:** Reverse transcription primer sequences of miRNAs

Gene	Genbank Accession	Reverse transcription primer sequences (5' → 3')
External Reference-RT		GTCGTATCCAGTGCAGGGTCCGAGGTATTCGCACTGGATACGACCAAGCT
hsa-miR-7-3p-RT	MIMAT0004553	GTCGTATCCAGTGCAGGGTCCGAGGTATTCGCACTGGATACGACTATGGC
hsa-miR-17-3p-RT	MIMAT0000071	GTCGTATCCAGTGCAGGGTCCGAGGTATTCGCACTGGATACGACCTACAA
hsa-miR-33a-5p-RT	MIMAT0000091	GTCGTATCCAGTGCAGGGTCCGAGGTATTCGCACTGGATACGACTGCAAT
hsa-miR-30a-3p-RT	MIMAT0000088	GTCGTATCCAGTGCAGGGTCCGAGGTATTCGCACTGGATACGACGCTGCA
hsa-miR-155-3p-RT	MIMAT0004658	GTCGTATCCAGTGCAGGGTCCGAGGTATTCGCACTGGATACGACTGTTAA

**Table 3:** Forward Primer for Real-Time PCR and Conditions

Gene	Forward Primer and Universal Primer (5' to 3')	Annealing (℃)
hsa-miR-7-3p-F	CGCGCAACAAATCACAGTCTG	60
hsa-miR-17-3p-F	GCGACTGCAGTGAAGGCACTT	60
hsa-miR-33a-5p-F	CGCGGTGCATTGTAGTTGC	60
hsa-miR-30a-3p-F	GCGCTTTCAGTCGGATGTTTG	60
hsa-miR-155-3p-F	CGCGCTCCTACATATTAGCA	60
External Reference-F	CACCGGGTGTAAATCAGCTTG	60
Universal Reverse Primer (micro-R)	AGTGCAGGGTCCGAGGTATT	60

**Table 4:** Real-Time PCR conditions of miRNAs

Conditions	Reaction systems (20 μL)
Sterile Distilled Water (SDW)	8.0 μL
Power SYBR® Green Master Mix	10.0 μL
Forward Primer (10 μM)	0.5 μL
micro-R (10 μM)	0.5 μL
cDNA	1.0 μL

Initial denaturation at 95℃ for 1 min, 40 Cycles (95 ℃ for 15 s, 60 ℃ for 25 s)

#### Dual luciferase assay

The bioinformatics assay results showed interactions between has-miR-155-3p and 3′-UTL of PPAR-α and between miR-17-3p and 3′-UTL of CYP7A1. A pmirGLO vector comprising 3′-UTR WT of PPAR-α, CYP7A1, or their 3′-UTR MUT was constructed in accordance with the literature method (Qianhua et al. 2023; Niu and Zhang 2023). The miRNA-19b-3p mimic or NC was co-transfected with PPARα WT or MUT, as well as CYP7A1 WT or MUT vector, in accordance with the instructions of Lipofectamine 3000 Transfection Reagent (Thermo Fisher, USA) in 293 T cells. After 48 h of incubation, each well of the cell culture plate was rinsed two times with PBS. Then, 250 µL of 1 × PLB lysis solution was added, and cells were lysed at room temperature. Then, Firefly and Renilla luciferase activities were measured using a dual luciferase reporter assay system (Promega, USA) in accordance with the operating instructions. The Firefly luciferase activity was analyzed by normalization against the Renilla luciferase activity.

### Data processing

The experimental data were measured three times in parallel, and the results were expressed as mean ± standard deviation. The data were statistically analyzed by using SPSS26.0, and one-way analysis of variance was used to analyze the significant differences among groups. *P* < 0.05 was considered as significant difference. *P* < 0.01 was considered as very significant difference.

## Results and analysis

### Monosaccharide composition of YCT-1, YCT-2 and YCT-3

The monosaccharide compositions of YCT-1, YCT-2, and YCT-3 are shown in Table 5, from which it can be seen that the three polysaccharide fractions were primarily composed of mannose, ribose, rhamnose, glucuronide, galacturonic acid, glucose, galactose, arabinose, and l-fucose. In addition, the monosaccharides with a high content of YCT-1 were mannose (3.209%), arabinose (8.016%), galactose (21.555%), glucose (58.274%), and xylose (3.141%). Moreover, the highly abundant monosaccharides in YCT-2 were mannose (3.286%), arabinose (18.034%), galactose (38.114%), glucose (30.454%), and rhamnose (3.217%). Then, the monosaccharides with a high content of YCT-3 were glucuronic acid (3.213%), arabinose (26.524%), galactose (52.394%), glucose (8.928%), and rhamnose (3.234%). These results showed that the monosaccharide composition of the three fractions was similar, but the molar ratios of these monosaccharides were very different, which indicate that the functional activities of these three polysaccharide fractions were also different.

**Table 5:** The monosaccharide composition of YCT-1, YCT-2 and YCT-3 (%)

Monosaccharide type	YCT-1	YCT-2	YCT-3
Guluronic acid	0.065	0.071	0.058
Mannose	3.209	3.286	2.156
Ribose	0.944	1.300	0.788
Rhamnose	2.073	3.217	3.234
Glucosamine	0.127	0.177	0.146
Glucuronic acid	1.199	2.033	3.213
Galacturonic acid	1.097	0.653	0.761
Galactose aminosus	0.026	0.041	0.028
Glucose	58.274	30.454	8.928
Galactose	21.555	38.114	52.394
Xylose	3.141	2.288	1.581
Arabinose	8.016	18.034	26.524
l-fucose	0.274	0.332	0.188

### Relative molecular masses of YCT-1, YCT-2 and YCT-3

**Fig. 1:**
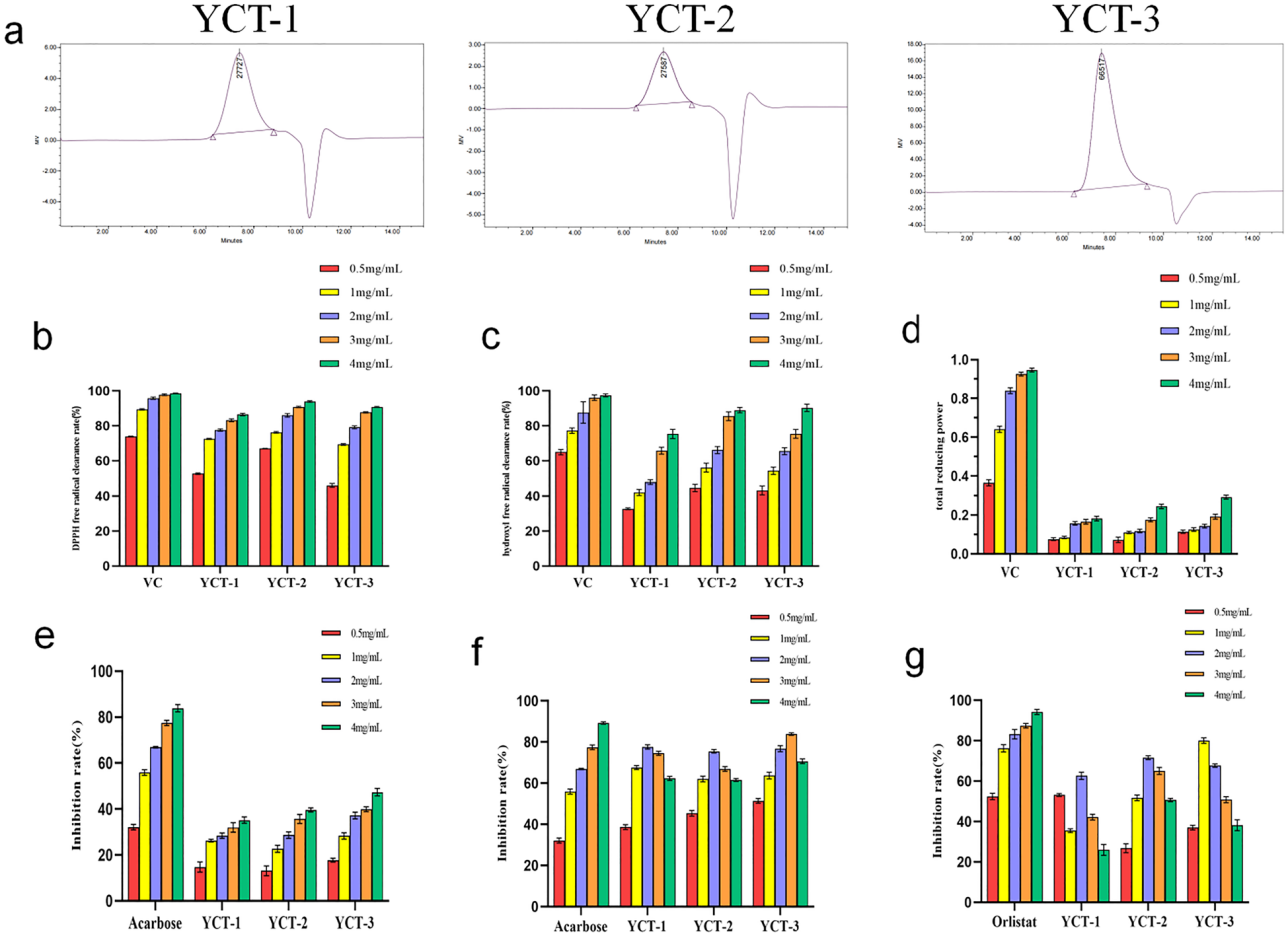
The molecular mass distribution and in vitro functional activities of tobacco polysaccharides fractions (YCT-1, YCT-2 and YCT-3). **a** Relative molecular mass distribution of YCT-1, YCT-2 and YCT-3; **b** DPPH free radical scavenging ability; **c** hydroxyl radical scavenging ability; **d** reducing power level, (VC was used as positive control); **e** The inhibitory effect on α-amylase activity; **f** The inhibitory effect on α-glucosidase activity, (Acarbose was used as positive control); **g** Inhibitory effect on pancreatic lipase activity (Orlistat was used as positive control)

The relative molecular mass distributions of YCT-1, YCT-2, and YCT-3 are shown in Fig. 1a, from which it can be seen that the weight-averaged relative molecular weights are 27,727, 27,587, and 66,517 Da, respectively. This result indicates that the three polysaccharide fractions obtained from the crude tobacco polysaccharides by cellulose chromatography columns have different relative molecular mass distributions.

### DPPH radicals were remarkably scavenged by tobacco polysaccharides

The DPPH radical scavenging activity of YCT-1, YCT-2, and YCT-3 is shown in Fig. 1b. As shown in the figure, the three fractions had an excellent scavenging ability for DPPH radicals with a good concentration dependence. The DPPH radical scavenging rate of YCT-2 at a concentration of 4 mg/mL was higher than that of the other two fractions. Overall, the maximum DPPH radical scavenging rates of YCT-1, YCT-2, and YCT-3 exceeded 85%, which was close to the VC value, indicating the good DPPH radical scavenging activities of YCT-1, YCT-2, and YCT-3.

### Hydroxyl radicals were scavenged by tobacco polysaccharides

Hydroxyl radicals existed in most cells, and the hydroxyl radical scavenging ability is an important means of evaluating antioxidant activity (Chen et al. 2010; Zhang et al. 2015; Di et al. 2021). The hydroxyl radical scavenging ability of YCT-1, YCT-2, and YCT-3 is shown in Fig. 1c. The hydroxyl radical scavenging rates of these three fractions also increased with the dependent concentration, which indicated their good quantitative-effect relationship. In addition, the maximum hydroxyl radical scavenging rate of YCT-2 reached 90.67%, which was higher than that of the other two fractions. Overall, the three fractions of tobacco polysaccharides have a good hydroxyl radical scavenging ability.

### Reducing power of tobacco polysaccharides was significantly observed

The reducing power is an index to evaluate the antioxidant capacity, which is obtained by the absorbance value to reflect the ability of Fe3 + to be reduced to Fe^2+^ (Shu-ship et al. 2014; Shi et al. 2008). As shown in Fig. 1d, the reducing power of YCT-1, YCT-2, and YCT-3 was increased with a good dose effect. The maximum reducing power of YCT-3 was higher than that of the other two fractions, but the overall difference in magnitude was not very significant (*P* > 0.05). Moreover, the overall level of the reducing power had a large gap when compared with VC, which indicated that the tobacco polysaccharide had a relatively weak reducing power, and this difference might be related to the low percentage of reductive monosaccharides.

### α-Amylase activity was slightly inhibited by tobacco polysaccharides

α-Amylase was used to hydrolyze starch before α-glucosidase hydrolyzes starch into glucose for absorption. Therefore, the inhibition of α-amylase can effectively reduce the starch digestibility, thereby inhibiting the sugar absorption to ameliorate hypoglycemia (Niu and Zhang 2023; Wang et al. 2020b). Acarbose is a hypoglycemic drug that can prevent the degradation of starch and other carbohydrates (Zhang et al. 2023, Gong and Lv 2018). In this study, the inhibitory effects of YCT-1, YCT-2, and YCT-3 on α-amylase were determined, and the results are shown in Fig. 1e. Within the range of experimental concentrations (0.5–4 mg/mL), YCT-1, YCT-2, and YCT-3 exhibited a weak inhibitory activity for α-amylase, but they had a good quantitative relationship. The maximum inhibition rate of α-amylase did not exceed 50%, and the maximum inhibition rate of α-amylase by acarbose (positive control) was 83.45%. When compared with acarbose, the inhibitory activities of YCT-1, YCT-2, and YCT-3 for α-amylase were relatively weak, so tobacco polysaccharides have a little inhibitory effect on α-amylase activity, but the activity is not strong.

### α-Glucosidase activity was evidently inhibited by tobacco polysaccharides

The inhibitory effects of YCT-1, YCT-2, and YCT-3 on α-glucosidase activity are shown in Fig. 1f. In the experimental concentration range (0.5–4 mg/mL), YCT-1, YCT-2, and YCT-3 showed significant inhibitory activities for α-glucosidase in a concentration-dependent manner. When the concentration of the fraction solution was increased to the maximum, the inhibition rate of α-glucosidase by YCT-1, YCT-2, and YCT-3 was close to that of acarbose. Therefore, YCT-1, YCT-2, and YCT-3 possessed a strong inhibitory activity for α-glucosidase, and it is expected to be developed into a natural and non-toxic hypoglycemic agent to replace traditional hypoglycemic drugs in the future.

### Pancreatic lipase activity was significantly inhibited by tobacco polysaccharides

The inhibition of the pancreatic lipase activity by YCT-1, YCT-2, and YCT-3 at different concentrations is shown in Fig. 1g. YCT-1 showed the maximum inhibitory activity (73.33%) at a concentration of 0.5 mg/mL. In addition, the maximum inhibitory activity of YCT-2 was observed at a concentration of 2 mg/mL, which reached 70.86%. Then, YCT-3 showed the highest inhibitory activity (80.54%) at 1 mg/mL. Overall, the highest inhibition rate of the three fractions for pancreatic lipase can reach more than 60%, which was slightly lower than the inhibition rate of a positive agent (orlistat), but tobacco polysaccharides had a better inhibitory activity for pancreatic lipase. However, with the increase of concentration, the inhibitory effect of tobacco polysaccharides on pancreatic lipase did not show a good concentration dependence.

### Lipid accumulation was significantly inhibited by tobacco polysaccharides in HepG-2 cells

**Fig. 2:**
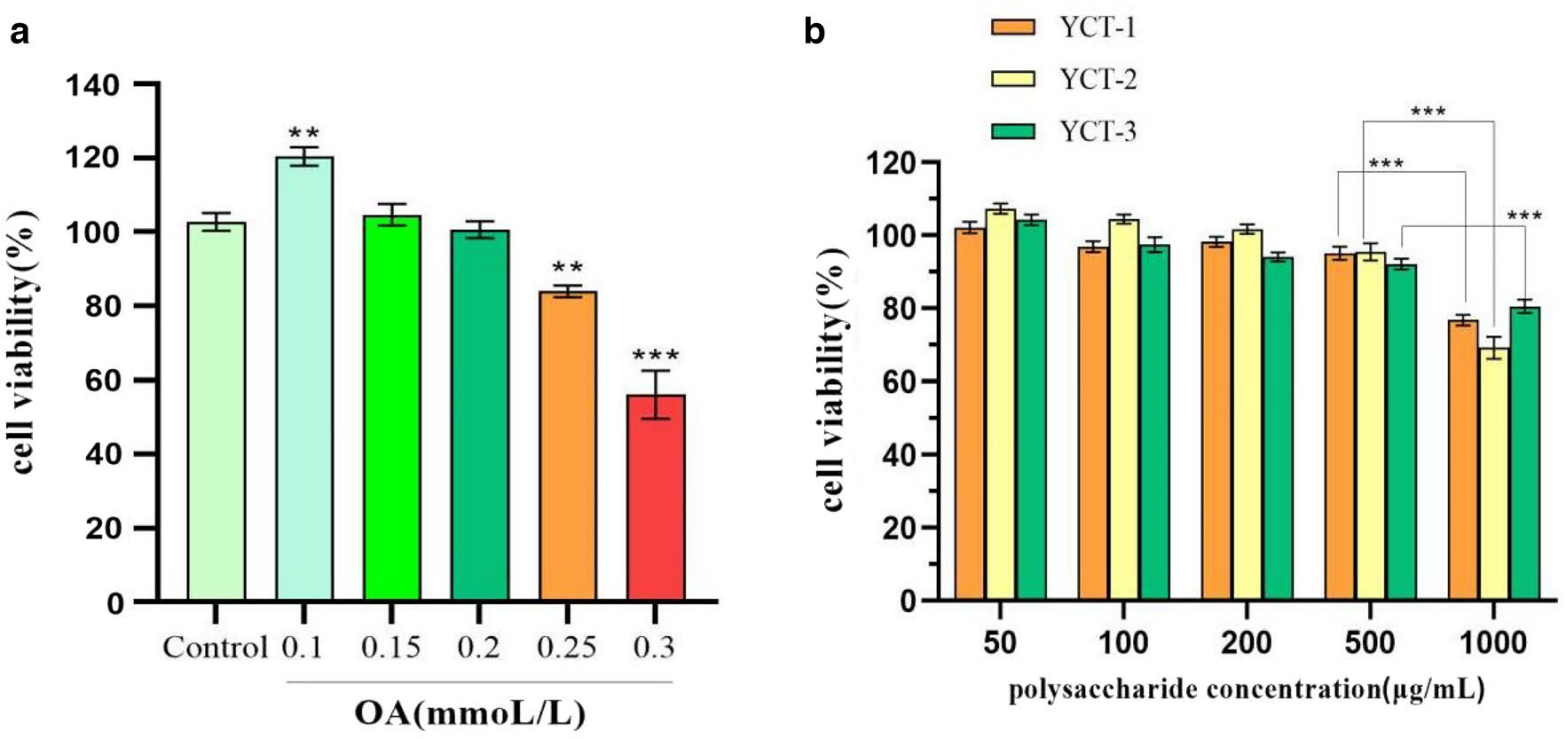
The survival rate of HepG-2 cells induced by OA and tobacco polysaccharides fractions (YCT-1, YCT-2 and YCT-3). **a** Effects of OA on survival rates of HepG-2 cells, ^**^P < 0.01, ^***^P < 0.001 vs Control group;** b** Effects of different tobacco polysaccharides fractions on the survival rate of HepG-2 cells, ^***^P < 0.001 for comparison between the two groups

The results of screening for appropriate model concentrations of oleic acid are shown in Fig. 2a. As the concentration of oleic acid was increased, the viability of HepG-2 cells was gradually decreased. When compared with the control group, the cell viability reached more than 99% at a concentration of 0.2 mmol/L, while the cell viability decreased to 83.37% when the concentration was 0.25 mmol/L, showing a significant difference (*P* < 0.01). Therefore, the optimal modeling concentration of oleic acid was determined to be 0.2 mmol/L. Then, the optimal concentration of tobacco polysaccharides with different molecular weights under the intervention of oleic acid was detected by using cell viability. The results are shown in Fig. 2b. When the concentration of the three fractions was 50, 100, 200, and 500 µg/mL, the cell viability was all above 95%, indicating that tobacco polysaccharides had a non-toxic effect on HepG-2 at the abovementioned concentrations. When the concentration was 1000 μg/mL, the cell viability of YCT-1, YCT-2, and YCT-3 was 75.41% ± 5.76%, 64.28% ± 5.24%, and 79.73% ± 5.54%, respectively, which indicated that tobacco polysaccharides at 1000 μg/mL can cause greater adverse effects on the cells. Therefore, the optimal concentration of the tobacco polysaccharides YCT-1, YCT-2, and YCT-3 was 500 μg/mL.

**Fig. 3:**
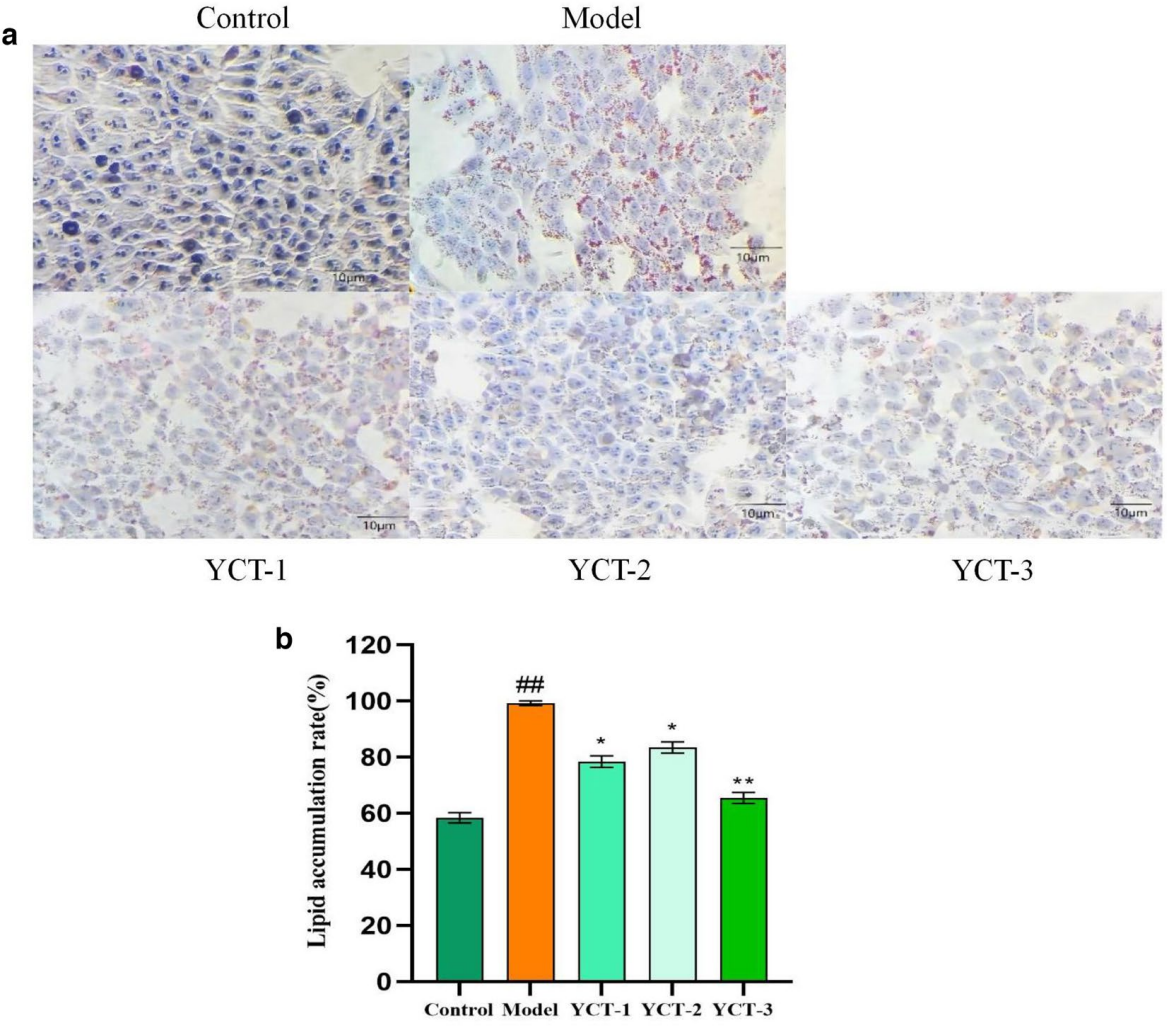
The effects of tobacco polysaccharides fractions on lipid accumulation in HepG-2 cells. **a** Observation of oil red O/hematoxylin staining of cells in different groups; **b** Effects of different polysaccharide components of tobacco on lipid deposition in OA-induced Hep G2 cells (oil red O staining), ^##^*P* < 0.01 vs Control group, ^*^P < 0.05, ^**^P < 0.01 vs Model group

After staining the cells with oil red O, lipid accumulation was observed and analyzed in different groups. As shown in Fig. 3a, when compared with the control group, evident orange–red lipid droplets spots were found in the cells of the model group, indicating the formation of a large number of lipid droplets in the cells and the successful establishment of the high-lipid model. When compared with the model group, the intracellular lipid accumulation was improved under the intervention of the tobacco polysaccharides YCT-1 (500 μg/mL), YCT-2 (500 μg/mL), and YCT-3 (500 μg/mL). Figure 3b shows the total intracellular lipid accumulation rate in each group, assuming that the lipid accumulation rate in the model group was 100%. The lipid accumulation rates of the YCT-1 (500 μg/mL), YCT-2 (500 μg/mL), and YCT-3 (500 μg/mL) groups accounted for 86.46%, 85.58%, and 78.85%, respectively. These results indicated that the three polysaccharide components can reduce lipid accumulation in HepG-2 cells, and the tobacco polysaccharide YCT-3 was superior to the other fractions.

### Lipid contents of TC, TG, LDL-c and HDL-c was significantly ameliorated by tobacco polysaccharides in HepG-2 cells

**Fig. 4:**
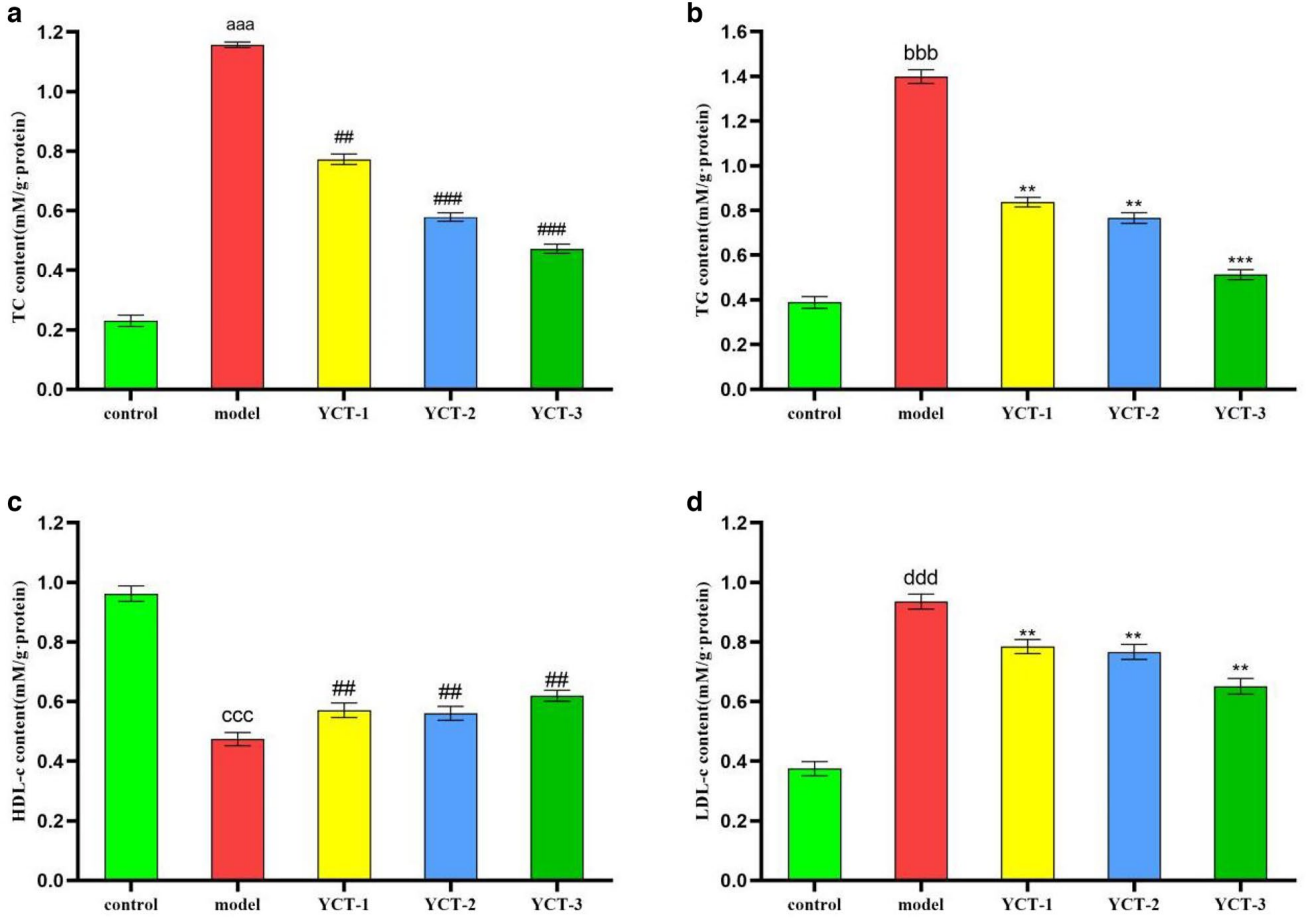
The effects of tobacco polysaccharides fractions on TC, TG, HDL-c and LDL-c content in HepG-2 cells. **a** TC content in HepG2 cell, ^aaa^*P* < 0.001 vs Control group, ^##^*P* < 0.01, ^###^*P* < 0.001 vs Model group; **b** TG content in HepG2 cell, ^bbb^*P* < 0.001 vs Control group, ^**^P < 0.01, ^***^P < 0.001 vs Model group;** c** HDL-c content in HepG2 cell, ^ccc^*P* < 0.001 vs Control group, ^##^*P* < 0.01 vs Model group; **d** LDL-c content in HepG2 cell, ^ddd^*P* < 0.001 vs Control group, ^**^P < 0.01 vs Model group

Figures 4a, b show the effects of tobacco polysaccharide fractions on total cholesterol (TC) and triglyceride (TG) contents induced by oleic acid in HepG-2 cells. As shown in the figure, the TC and TG contents in the model group were significantly higher than those in the control group, which indicated that the model establishment was successful. When compared with the model group, the TC and TG contents in the experimental groups of YCT-1 (500 µg/mL), YCT-2 (500 µg/mL), and YCT-3 (500 µg/mL) were significantly decreased. In addition, the TC and TG contents in the YCT-1 group were decreased by 32.84% and 40.04%, respectively. Moreover, the TC and TG contents in the YCT-2 group decreased by 49.91% and 44.99%, and the TC and TG contents in the YCT-3 group were decreased by 58.81% and 63.32%, respectively. Therefore, YCT-3 was more effective in lowering TC and TG contents when compared with the other two tobacco polysaccharide fractions.

Figures 4c, d show the effect of the tobacco polysaccharides YCT-1, YCT-2, and YCT-3 on high-density lipoprotein cholesterol (HDL-c) and low-density lipoprotein cholesterol (LDL-c) content in HepG-2 cells. As shown in the figure, the HDL-c content in the model group was significantly lower than that of the control group, whereas the LDL-c content was significantly higher than that of the control group. When compared with the model group, the experimental sample groups of YCT-1 (500 µg/mL), YCT-2 (500 µg/mL), and YCT-3 (500 µg/mL) showed that the HDL-c content was increased with different degrees, whereas the LDL-c content was reduced with different degrees. In particular, the HDL-c content was increased by 20.68%, 19.41%, and 27.43% in the YCT-1, YCT-2, and YCT-3 groups, respectively. Moreover, the LDL-c content was decreased by 16.04%, 17.86%, and 30.27% in the YCT-1, YCT-2, and YCT-3 groups, respectively. Therefore, the abovementioned results indicated that the tobacco polysaccharide YCT-3 was more effective in modulating HDL-c and LDL-c contents in HepG2 cells.

### Expression levels of lipid metabolism-related genes were evidently regulated by tobacco polysaccharides in HepG-2 cells

**Fig. 5:**
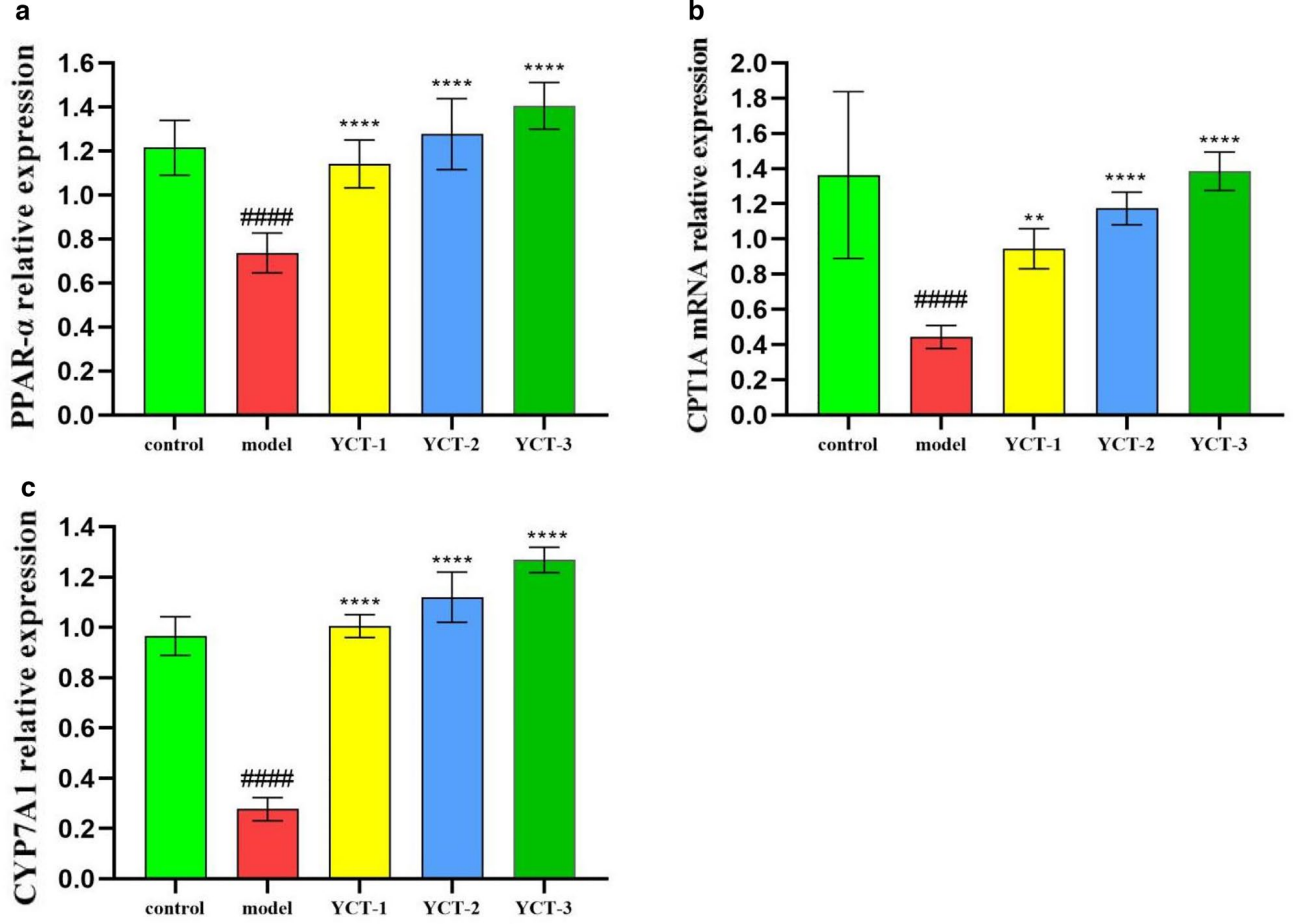
The effects of tobacco polysaccharides fractions on mRNA levels of lipid-regulated genes in HepG-2 cells. **a** Relative mRNA expression of PPAR-α gene in HepG2 cells; **b** Relative mRNA expression of CPT1A gene in HepG2 cells; **c** Relative mRNA expression of CYP7A1 gene in HepG2 cells. ^####^*P* < 0.0001 vs Control group, ^**^P < 0.01, ^****^P < 0.0001 vs Model group

PPAR-α, a member of the nuclear receptor superfamily, is activated in the liver by natural ligands such as fatty acids and their metabolites and is involved in regulating the expression level of lipid metabolism–related genes such as fatty acid oxidation and cholesterol metabolism in the liver. CYP7A1, a rate-limiting enzyme for cholesterol catabolism and bile acid synthesis, belongs to the liver-specific microsomal P450 superfamily and plays an important role in maintaining cholesterol homeostasis. CPT-1A is a key enzyme controlling fatty acid uptake and oxidation in the mitochondria, and it controls the rate of fatty acid oxidation by converting acyl coenzyme A to acyl carnitine (Gong and Lv 2018; Xu et al. 2010). In this experiment, the mRNA expression level of PPAR-α, CPT-1A, and CYP7A1 in HepG-2 cells was detected by RT-qPCR, and the results are shown in Fig. 5a–c. As shown in the figures, the mRNA expression level of PPAR-α, CPT1A, and CYP7A1 in the model group was significantly decreased as compared to that in the normal group (*P* < 0.01). When compared with the model group, the mRNA expression level of PPAR-α, CPT1A, and CYP7A1 in the cells of YCT-1 (500 μg/mL), YCT-2 (500 μg/mL), and YCT-3 (500 μg/mL) was increased with different degrees. When compared with the control group, the relative expression level of PPAR-αmRNA in the YCT-1 (500 μg/mL), YCT-2 (500 μg/mL), and YCT-3 (500 μg/mL) groups had no significant difference (*P* > 0.05). The relative expression level of CPT1AmRNA in the YCT-1 (500 μg/mL) group was significantly different from that in the control group (*P* < 0.05), but no significant difference was found in the YCT-2 (500 μg/mL) and YCT-3 (500 μg/mL) groups when compared with the control group (*P* > 0.05). The relative expression level of CYP7A1 mRNA in the YCT-2 (500 μg/mL) and YCT-3 (500 μg/mL) groups was significantly different from that in the control group (*P* < 0.01), but no significant difference was found in the YCT-1 (500 μg/mL) group compared with the control group (*P* > 0.05). Compared with YCT-1 and YCT-2, YCT-3 was more effective in up-regulating the mRNA expression level of PPAR-α, CPT1A, and CYP7A1. These results indicated that the three tobacco polysaccharide fractions can regulate the lipid metabolism in HepG2 cells by modulating the mRNA expression level of lipid metabolism–related genes, and the regulatory effect of YCT-3 was more pronounced.

### Extraction and identification of exosomes from HepG-2 cells

**Fig. 6:**
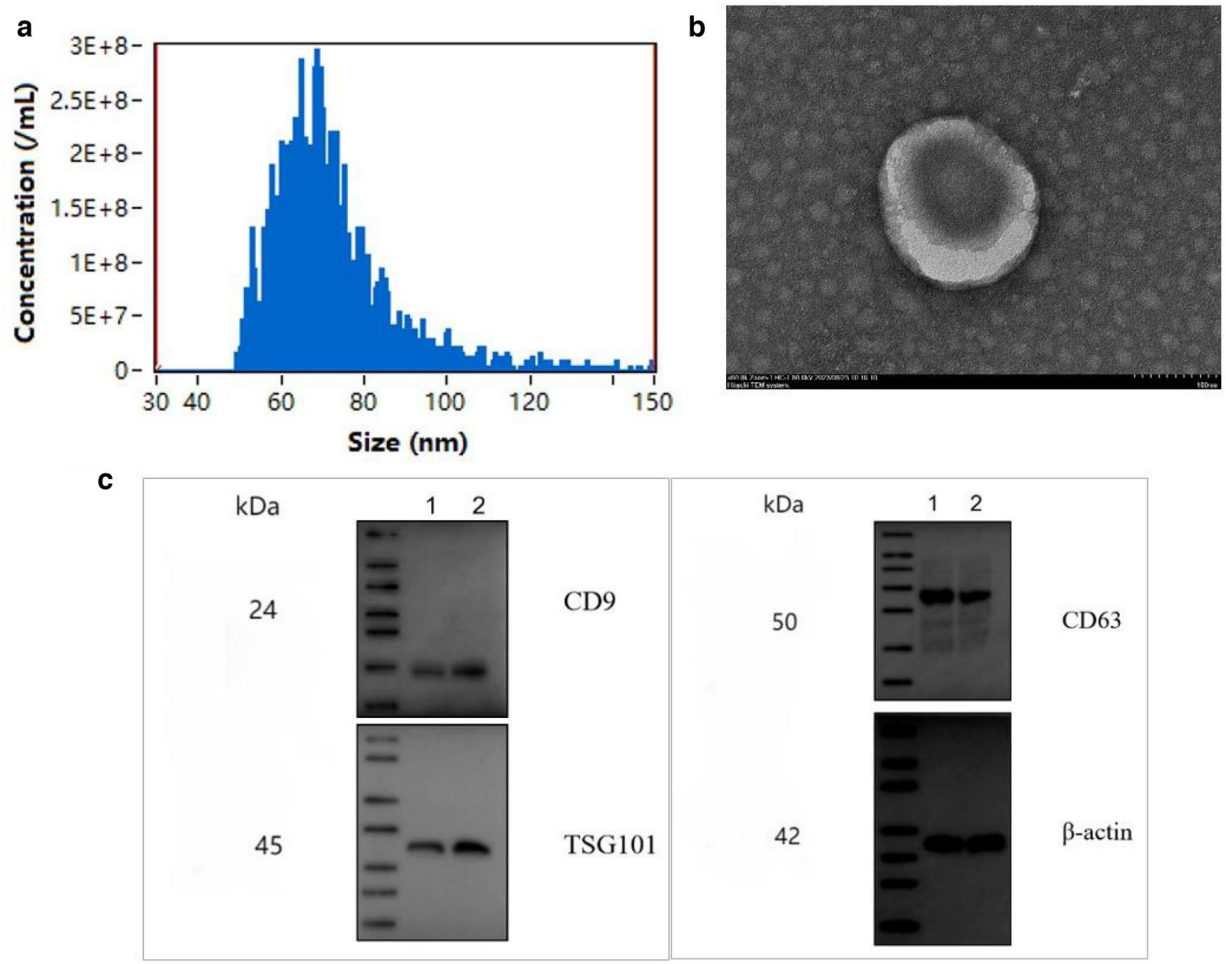
The identification of exosomes from HepG-2 cells. **a** Particle-size analysis showing that the particle size of each exosome ranged from 50 to 150 nm;** b** HepG2 cell exosomes of different groups observed by TEM at the scale of 200 nm; **c** Expression levels of CD9, CD63, and TSG101 determined by Western blot

MicroRNA (miRNA) is a non-coding small-molecule RNA that can exist in active form in exosomes, transfer to recipient cells with exosome secretion, and participate in various aspects of metabolic regulation through unique transcription (Xu, et al. 2010; Zhou and Huang 2023a, b; Hou et al. 2021). The size distribution of exosomes was measured by using a Flow Nano-Analyzer instrument (Fig. 6a). The morphology of exosomes was observed by using a TEM (Fig. 6b). In addition, CD9, CD63, and TSG101 proteins in exosomes were identified by Western blot (Fig. 6c). As shown in Fig. 6a, b, the exosomes derived from HepG-2 showed the morphology of small cup-shaped round vesicles with a double-membrane structure. The detected results showed that the particle size of exosomes ranged from 50 to 150 nm. As shown in Fig. 6c, the detection results showed that the specific marker proteins CD9, CD63, and TSG101 of HepG2 cell exosomes were expressed, which confirmed that the detected substance was indeed HepG2 cell exosomes.

### YCT-3 significantly regulated lipid metabolism by the interaction between mRNAs and miRNAs

**Fig. 7:**
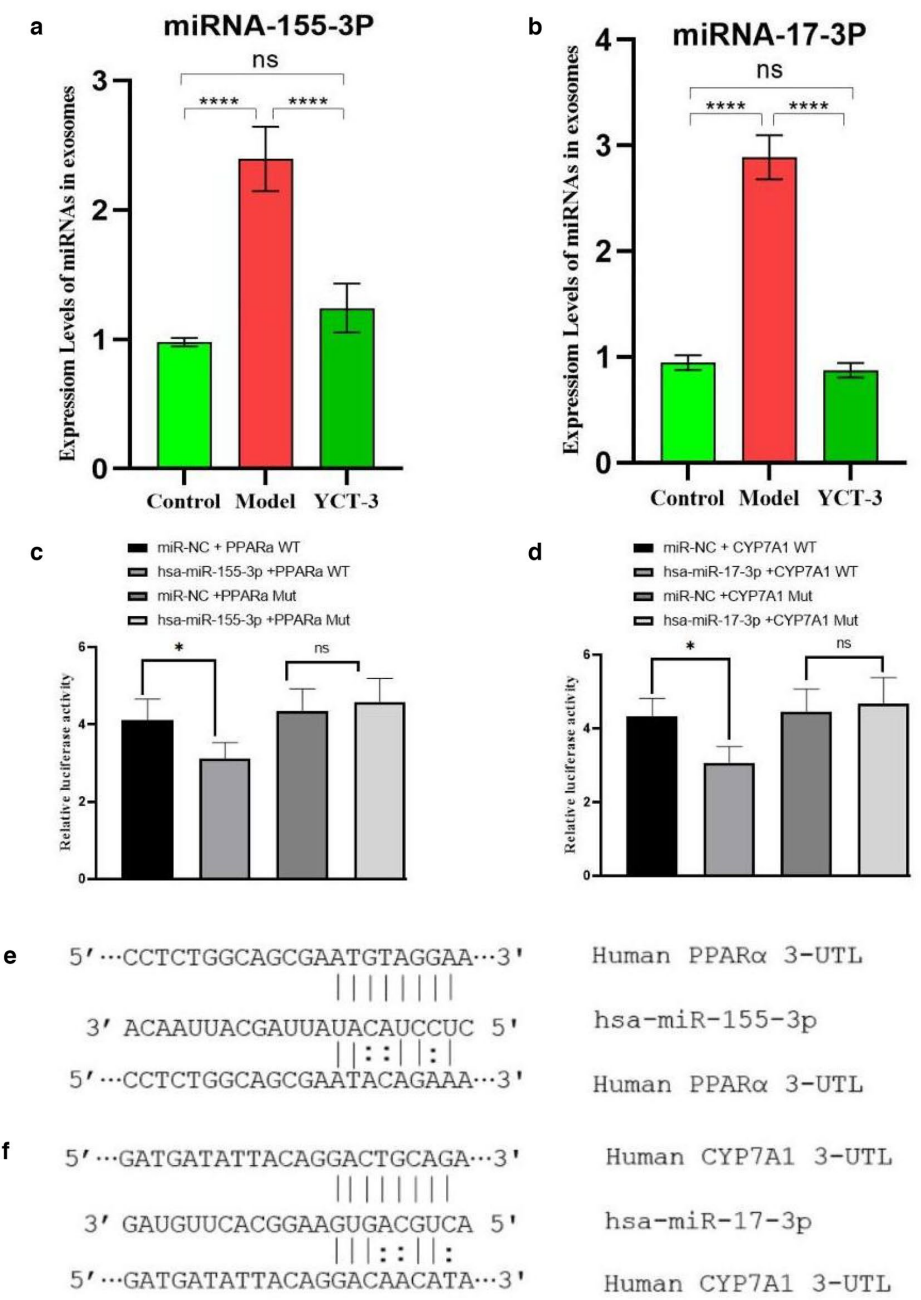
The effects of tobacco polysaccharides fractions on exosomal miRNAs expression levels and interaction between miRNA and mRNA. **a** The expression levels of HepG2 cell exosome miR-155-3p were detected by RT-qPCR;** b** The expression levels of HepG2 cell exosome miR-17-3p were detected by RT-qPCR;** c** The relative fluorescence values after transfection and mutation, which suggested that significant interaction existed between miR-155-3p and target gene PPAR-α; **d** The relative fluorescence values after transfection and mutation, which suggested that significant interaction existed between miR-17-3p and target gene CYP7A1;** e** The possible interaction sites for miR-155-3p and target gene PPARα were predicted;** f** The possible interaction sites for miR-17-3p and target gene CYP7A1 were predicted

The expression levels of miR-155-3p and miR-17-3p in the exosomes from different groups are shown in Fig. 7a, b. MiR-155-3p and miR-17-3p expression levels were significantly up-regulated in the model group compared with the control group. Compared with the model group, miR-155-3p and miR-17-3p levels were significantly down-regulated in the YCT-3 group. Then, the possible interaction sites between miR-155-3p and its target gene PPAR-α as well as between miR-17-3p and its target gene CYP7A1 were predicted by TargetScan (Fig. 7e, f). Their interactions were further detected by constructing reporter plasmids for miR-155-3p and PPAR-α mRNA as well as for miR-17-3p and CYP7A1 mRNA with dual luciferase reporter gene assays. The results showed that miR-155-3p and miR-17-3p significantly reduced the levels of PPAR-α WT and CYP7A1 WT in 293 T cells (*P* < 0.05, Fig. 7c, d), whereas the levels of PPAR-α MUT and CYP7A1 MUT were not affected. Thus, the alleviating effect of YCT-3 on abnormal lipid metabolism may be related to the regulation of the miRNA signaling pathway.

## Conclusions

In the present study, the crude tobacco polysaccharide was obtained by hot water extraction, and purified and separated by DEAE cellulose chromatography column, and three purified polysaccharide fractions, YCT-1, YCT-2 and YCT-3, were finally obtained, then their physicochemical properties and in vitro hypoglycemic and hypolipidemic activities were analyzed. The results showed that the three tobacco polysaccharide fractions had different relative molecular masses and monosaccharide compositions, and all of them possessed well antioxidant activity and hypoglycemic and hypolipidemic activity. The results of cellular experiments showed that tobacco polysaccharides were capable of regulating the expression level of lipid metabolism-related gene by influencing the expression of miRNAs in cellular exosomes, and then ameliorated the excessive accumulation of intracellular lipids. Based on the antioxidant activity and hypoglycemic and hypolipidemic activity of tobacco polysaccharides, it is expected to be developed into a new type of plant-derived polysaccharide health care product or a new type of drug for the prevention and treatment of hyperlipidemia in the future, so as to realize the comprehensive utilization of tobacco resources and alleviate the environmental pollution caused by the treatment of tobacco wastes.

## Data Availability

The datasets used and/or analyzed during the current study are available from the corresponding author upon reasonable request.

## Abbreviations

CPT-1A: Carnitine palmitoyltransferase-1A
CYP7A1: Cytochrome P450 7A1
PPAR-α: Peroxisome proliferators activated receptors α
YCT-1: Tobacco polysaccharide fraction 1
YCT-2: Tobacco polysaccharide fraction 2
YCT-1: Tobacco polysaccharide fraction 3
HDL-C: High-density lipoprotein cholesterol
LDL-C: Low-density lipoprotein cholesterol
TC: Total cholesterol
TG: Triglyceride
PMP: 1-Phenyl-3-methyl-5-pyrazolone
